# Repeatability and reproducibility of a new classification for measuring primary stability of dental implants based on progressive insertion torque value: An analytical observational study

**DOI:** 10.4317/jced.63321

**Published:** 2025-12-30

**Authors:** José Rosas-Díaz, Nancy Córdova-Limaylla, Maria Eugenia Guerrero, Jerson Palomino-Zorrilla, Rocío Álvarez-Medina, César Cayo-Rojas

**Affiliations:** 1 School of Stomatology, Universidad Privada San Juan Bautista, Lima, Peru; 2 Faculty of Stomatology, Universidad Peruana Cayetano Heredia, Lima, Peru; 3 Medico Surgical Department, Faculty of Dentistry, Universidad Nacional Mayor de San Marcos, Lima, Peru; 4 Faculty of Dentistry, Universidad de San Martin de Porres, Lima, Peru

## Abstract

**Background:**

Assessing primary stability is critical to predicting dental implant success, yet existing assessments often rely on subjective judgment. This study presents and validates a classification system based on the progressive insertion torque value (PITV) to provide objective, reproducible parameters for guiding prosthetic loading protocols.

**Material and Methods:**

An analytical observational study was conducted on 1,250 implant torque-curve interpretations. Primary stability was classified into three types according to final insertion torque (IT): high (50 Ncm), moderate (30 to &lt;50 Ncm), and low (&lt;30 Ncm). Types I and II included four PITV subtypes (A-D), while Type III included six subtypes (A-F) based on curve trajectory patterns. Twenty-five dentists were trained in curve interpretation, and intra- and inter-examiner agreement was assessed using Cohen's and Fleiss' kappa statistics and prevalence- and bias-adjusted kappa (PABAK). Predictive validity was assessed using receiver operating characteristic (ROC) analysis (area under the curve [AUC] with 95% confidence intervals). Calibration (intercept and slope) and the Brier score were also computed.

**Results:**

Intraexaminer agreement was almost perfect in both general dentists and implant specialists (k = 0.82-0.95). Interexaminer agreement was also almost perfect (general dentists: k = 0.81; specialists: k = 0.89). Overall agreement was k = 0.84 (p &lt; 0.001). The classification showed moderate predictive discrimination (AUC = 0.69, 95% CI: 0.56-0.81).

**Conclusions:**

The classification of primary stability based on the progressive insertion torque value in edentulous maxillary ridges showed high intraexaminer and interexaminer reliability in interpreting torque-time curves. Its clinical utility, predictive performance, and prosthetic loading thresholds were not assessed in this study and warrant prospective evaluation.

## Introduction

During the planning of dental implant treatment, it is of paramount importance to determine the bone quality of the edentulous ridges. This allows for an accurate diagnosis of the implant bed conditions, which in turn supports clinical decision-making and optimizes outcomes (1 - 5). Bone quality represents one of the most significant determinants in the prediction of early dental implant failures. Consequently, the Lekholm and Zarb classification is frequently employed in cone-beam computed tomography (CBCT) for this evaluation (6 , 7). In this context, it has been reported that the mean survival rate of dental implants placed in the maxillae with type I-IV bone was 97.6%, 96.2%, 96.5%, and 88.8%, respectively (3). Additionally, several clinical studies have observed that implants placed in the mandible exhibit higher survival rates compared with those placed in the maxilla, particularly in the posterior region (3 - 5). To enhance the evaluation of bone quality, which considers combinations of cortical and cancellous bone, several modifications have been proposed to the subjective radiographic evaluation by Lekholm and Zarb. Among these is the proposal by Rosas et al., which introduces two new types (V and VI), subdivides type II into three subtypes (II-A, II-B, and II-C), and type III into two subtypes (III-A and III-B). These modifications are based on an evaluation of bone quality with respect to cortical thickness, the visibility and amount of bone trabeculae, and the size of the medullary spaces in the cancellous bone (2 , 6). For decades, it has been suggested that true bone quality is determined clinically-rather than radiographically-by tactile sensation at the time of osteotomy preparation. However, this method remains subjective and poorly reproducible. In the ongoing effort to standardize evaluation criteria that support more effective treatment planning, progressive insertion torque, measured using a digital torque meter at the time of implant insertion, could be incorporated as a quantitative measure and even as a prognostic indicator of osseointegration. This would involve evaluating the implant pathway from the moment it begins to enter the bone until it reaches the final crestal position (8 , 9). Along this trajectory, several factors directly related to bone quality-such as cortical thickness, trabecular thickness, and the size of the medullary spaces-can influence the mechanical interlocking of the implant (10 , 11). In contrast to the conventional approach of considering only the final torque, analyzing the full progressive insertion torque curve provides a more comprehensive understanding of the factors influencing treatment success. It is well established that the final torque provides an indication of implant anchorage. Strong anchorage correlates with favorable clinical outcomes, influencing primary and secondary stability as well as considerations for immediate loading. Consequently, objective clinical evaluation of this process, enabled by digital torque meters, is crucial, since there are currently no classification systems based on objective parameters to guide decision-making and ensure predictable long-term outcomes. Historically, only the final torque value has been used as a parameter to measure primary stability, rather than the torque-time curve. Common clinical classifications indicate that torque is low when it is &lt;20 Ncm and is typically associated with low-density bone or an overly wide surgical bed, for which deferred loading is recommended. Moderate torque (20-35 Ncm), generally considered acceptable and safe for most cases, allows conventional protocols and, in selected cases, early loading. High torque (35-45 Ncm) reflects good primary stability and is the recommended range for immediate loading of prostheses, whereas excessive torque (&gt;45 Ncm) may risk bone necrosis due to over-compression (12 , 13). Accordingly, the objective of this study was to propose a novel classification for measuring the primary stability of dental implants based on the progressive insertion torque value (PITV). This classification aims to provide clinical parameters that facilitate the success of primary and secondary stability (osseointegration) and inform loading protocols, while demonstrating a high degree of repeatability and reproducibility.

## Material and Methods

**Figure 1:**
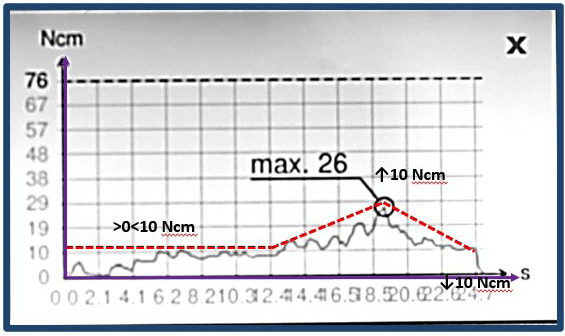
Classification of torque curves according to their trajectory: Horizontal—no decline greater than 10 N•cm along the entire path, with a predominance of horizontal alignment; Ascending—increase of more than 10 N•cm over time, with a predominance of vertical rise toward the end; Descending—decrease of more than 10 N•cm over time, with a predominance of vertical decline.

1. Study design An observational, retrospective, and analytical study was conducted. This report was written in accordance with the Strengthening the Reporting of Observational Studies in Epidemiology (STROBE) guidelines for observational studies. 2. Population and sample selection The study population comprised 150 insertion torque-time curve records obtained at the Centro de Investigación Estomatológica Peruana (CIOP) in Lima, Peru, between 2019 and 2022. All implant osteotomies were prepared by a single operator, a specialist in Comprehensive Oral Implantology, using the manufacturer's drilling protocol. Standardized implants (SIN, Epikut; São Paulo, Brazil; diameters 3.5, 3.8, 4.5, and 5.0 mm; lengths 8.5, 10.0, 11.5, 13.0, and 15.0 mm) were used in all 150 cases. The macrodesign was uniform (conical walls) and the implants were placed with 0.2 mm undersizing relative to the prepared osteotomy. All implants had a double acid-etched surface with a 20 nm hydroxyapatite coating. From this database, we drew a simple random sample of 50 curves exclusively for the repeatability and reproducibility analysis; these 50 curves were not used to develop the classification. Although nominal implant diameters and lengths are broadly comparable across manufacturers, the use of a single motor-implant system and a fixed osteotomy protocol in this study limits external validity; therefore, multiplatform and multi-operator validation is warranted. Bone quality was classified as types I, II-A, II-B, II-C, III-A, III-B, IV, and V according to the Lekholm and Zarb classification as modified by Rosas et al. Patients had no local bone pathology or systemic disease. Sequential drilling was performed in all 150 cases with standardized diameters: spear drill at 1,200 rpm, followed by a 2.0-mm drill at 1,200 rpm, and subsequent drills-according to the final implant diameter-at 800 rpm. Preparation depths were standardized using a safe drill with depth stops for apicocoronal accuracy. Bone bed preparation and final implant placement torque measurements (Ncm) were performed with a W&amp;H Implantmed SI-1023 motor (max 40,000 rpm; 220-240 V AC, 50-60 Hz; W&amp;H, Austria), and with a digital torque wrench calibrated to a maximum torque of 80 Ncm. Data were extracted from medical records, including bone-quality category, surgical protocol, implant length and diameter, surface treatment (nanostructured, HA-coated), systemic health status, final torque, maximum torque, and the full torque-time curve at completion of surgery. Each torque curve was analyzed and classified according to final torque value and curve shape, with the midpoint corresponding to 50% of the implant insertion depth. Torque curves were evaluated at the beginning, mid-procedure, and at the end of implant placement, yielding a total of 1,250 interpretations. The sample size was calculated using Epidat 4.2, applying the formula for frequency estimation with a known sampling frame (N = 150), assuming p = 0.95 and an absolute precision of 0.05 (6). A total of 50 torque-curve records were selected by simple random sampling without replacement, performed by a dental implant specialist who was not involved in the calibration process. The selection criteria were as follows: Inclusion criteria Records of insertion torque-time (progressive) curves (Ncm) obtained during dental implant placement from patients who provided written informed consent for the research use of their data. Records of insertion torque-time (progressive) curves (Ncm) obtained during dental implant placement from healthy patients or those with controlled systemic conditions not affecting bone metabolism. Records of insertion torque-time (progressive) curves (Ncm) obtained during dental implant placement from patients who underwent surgery performed by a dental implant specialist. Exclusion criteria Records of insertion torque-time (progressive) curves (Ncm) obtained during dental implant placement from patients who experienced early or late implant loss. Records of insertion torque-time (progressive) curves (Ncm) obtained during dental implant placement from patients who required immediate implant placement or received implants in areas with bone defects or active pathology. 3. Classification of primary stability of dental implants based on progressive insertion torque value (PITV). This proposal is based on an objective evaluation of bone quality as reflected by the resistance offered by the cortical and trabecular bone during dental implant insertion, expressed as insertion torque (Ncm). The insertion torque provides a curve in Ncm in which the x-axis represents time (s) and the y-axis represents torque (Ncm). The classification of bone quality was determined by calculating the final insertion torque (FIT; Ncm) and the shape of the curve in its initial segment-which corresponds to the first third of insertion-and its terminal segment-which corresponds to the final position of the implant platform at the crestal cortical level (Fig. 1).

1

**Figure 2:**
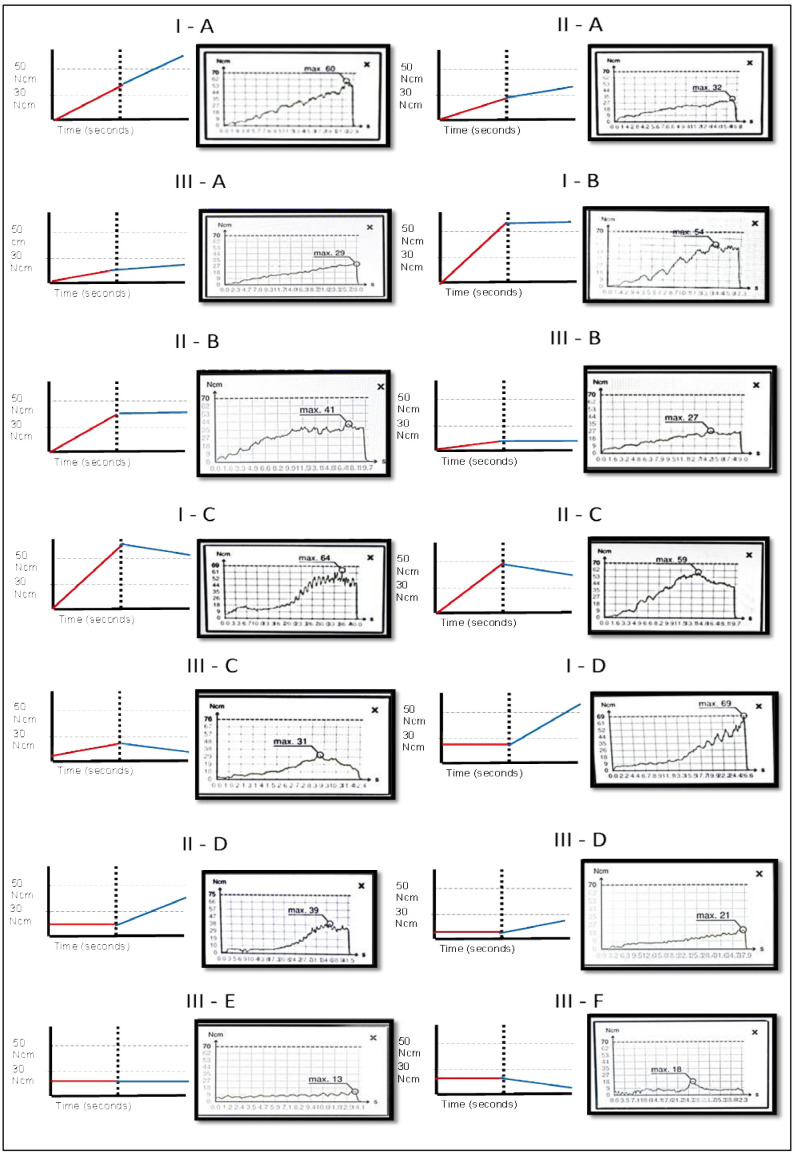
Classification of primary stability of dental implants based on progressive insertion torque value in N•cm at the moment of inserting the dental implant in the bone bed. Type I, Type II, Type III and Subtypes A, B, C, D, E and F.

The values were pragmatically classified into the following categories: Primary stability Type I: High final torque 50 Ncm. Subtype A, B, C and D. Primary stability Type II: Moderate final torque 30 Ncm, &lt;50 Ncm. Subtype A, B, C and D. Primary stability Type III: Low final torque &lt;30 Ncm. Subtype A, B, C, D, E and F. High torque can be subdivided into high (50-60 Ncm) and very high (60 Ncm), and low torque into low (&lt;30 to 20 Ncm) and very low (&lt;20 Ncm). Each stability tier is assigned to a subtype (letters A-F) according to the early and late behavior of the torque-time curve. For operational consistency, we pre-specified a ±10 Ncm tolerance to classify segments as follows: ascending if the net change () over the segment exceeds +10 Ncm, descending if is below 10 Ncm, and horizontal if || 10 Ncm. This ±10 Ncm band was chosen pragmatically to buffer the instrument's measurement resolution and small intra-curve variations, while also avoiding crossings of common clinical decision thresholds around 30 and 50 Ncm. Segment behavior was computed over the first and final third of the insertion path (Fig. 1). Types of curves according to the combination of ITV and PITV Type I-A: High final torque 50 Ncm; initial segment: ascending; terminal segment: ascending. Type I-B: High final torque 50 Ncm; initial segment: ascending; terminal segment: horizontal. Type I-C: High final torque 50 Ncm; initial segment: ascending; terminal segment: descending. Type I-D: High final torque 50 Ncm; initial segment: horizontal; terminal segment: ascending. Type II-A: Moderate final torque 30 Ncm and &lt;50 Ncm; initial segment: ascending; terminal segment: ascending. Type II-B: Moderate final torque 30 Ncm and &lt;50 Ncm; initial segment: ascending; terminal segment: horizontal. Type II-C: Moderate final torque 30 Ncm and &lt;50 Ncm; initial segment: ascending; terminal segment: descending. Type II-D: Moderate final torque 30 Ncm and &lt;50 Ncm; initial segment: horizontal; terminal segment: ascending. Type III-A: Low final torque &lt;30 Ncm; initial segment: ascending; terminal segment: ascending. Type III-B: Low final torque &lt;30 Ncm; initial segment: ascending; terminal segment: horizontal. Type III-C: Low final torque &lt;30 Ncm; initial segment: ascending; terminal segment: descending. Type III-D: Low final torque &lt;30 Ncm; initial segment: horizontal; terminal segment: ascending. Type III-E: Low final torque &lt;30 Ncm; initial segment: horizontal; terminal segment: horizontal. Type III-F: Low final torque &lt;30 Ncm; initial segment: horizontal; terminal segment: descending (Fig. 2).

2

**Figure 3:**
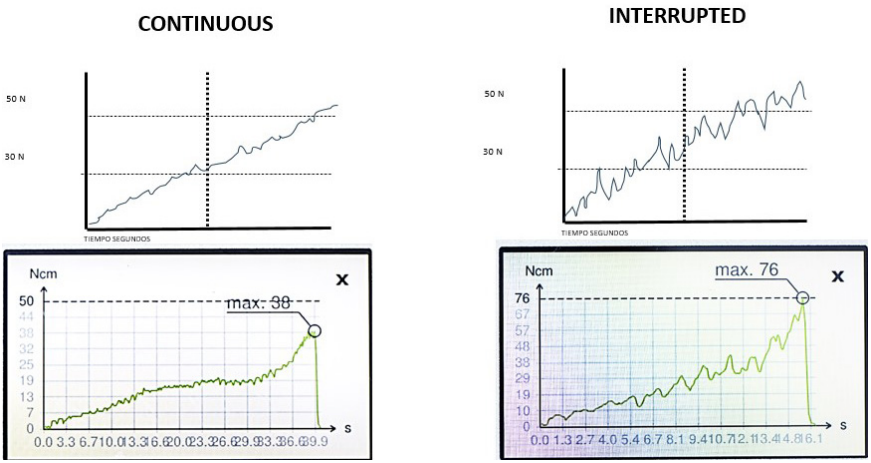
Dental implants insertion torque curve subtypes. When peaks and valleys with a drop of less than 5 N•cm predominate, it is considered a continuous curve and is related to small marrow spaces. When peaks and valleys with drops greater than 5 N•cm predominate, it is considered a discontinuous or interrupted curve, related to wide marrow spaces and very fine bone trabeculae, where implant stability drops and rises again irregularly.

Some of the curves exhibited a linear behavior, while others exhibited a zigzag pattern with pronounced peaks and valleys and abrupt changes of more than 5 Ncm along their course. These continuous and discontinuous patterns were associated with the presence of large versus narrow medullary spaces in the trabecular bone, as well as thinner versus thicker trabeculae, which produced the sharp decreases or increases in stability (Fig. 3).

3

4. Preparation and Validation A team of five dental implantology specialists, each with more than 10 years of experience, developed a new bone-quality classification based on the insertion torque-time curve. Subsequently, five additional specialists evaluated the clarity, objectivity, timeliness, organization, sufficiency, intentionality, consistency, coherence, and methodology of the classification using an ordinal scale with the following categories: poor (0.00-0.20), fair (0.21-0.40), good (0.41-0.60), very good (0.61-0.80), and excellent (0.81-1.00). Content validity, quantified by Aiken's V, was V=0.97 (95% CI, 0.95-0.98). After finalizing the classification, 13 implant specialists and 12 general dentists (all with &gt;10 years' experience), external to this study, were trained in its application. The 25 dentists then participated in intra- and inter-examiner calibration exercises. They analyzed 50 insertion torque-time curves (Ncm) that an independent researcher (who did not participate in the calibration) shared in real time via Microsoft Teams at two sessions within seven days for each participant. At each session, the 25 participants independently classified bone quality according to the training they had received. None of the 50 curves were used to develop the classification. An independent researcher stored the data, de-identified participants, and tabulated results; a single-blinded biostatistician performed the analysis. All curve recordings were obtained using the same Implantmed O4601 surgical motor (W&amp;H, Burmoos, Austria). 2.5 Statistical repeatability and reproducibility analysis Data were imported from Microsoft Excel 2019 into SPSS version 28.0 (IBM Corp., Armonk, NY, USA). To quantify intra-examiner agreement and potential interpretation bias in classifying bone quality from the insertion torque-time curve (Ncm), we used Cohen's and interpreted results according to the Landis and Koch ordinal categories ("poor" to "almost perfect") (6 , 14 , 15). Similarly, inter-examiner agreement was assessed with Fleiss' , using the same categories. For all statistical analyses, the significance threshold was set at p&lt;0.05 (Table 1).

Table 1

An a priori sample-size calculation for Cohen's was performed using lrstat::getDesignAgreement (R 4.3.1), testing H0: 0.40 vs H1: 0.70 with = 0.05 (one-sided) and an expected marginal distribution based on our benchmark set. This yielded a required n = 24 curves (power 1 = 0.807). The final agreement dataset comprised n = 50 curves. To address prevalence- and bias-related effects, we pre-specified reporting PABAK alongside and report the subtype distribution. For inter-examiner agreement, we also report Fleiss' together with overall percent agreement and Gwet's AC1 (nominal, multi-rater; with finite-sample correction). To assess predictive performance, a retrospective set of 150 curves with 4-6 months of follow-up was analyzed for osseointegration (success/failure). Receiver operating characteristic (ROC) analysis was used to estimate AUC with 95% CIs, and calibration (intercept and slope) as well as the Brier score were computed. 2.6 Human Ethics and Consent to Participate This study adhered to the principles of the Declaration of Helsinki (16), which pertain to the principles of beneficence, justice, autonomy, and non-maleficence. Furthermore, it was approved by the Institutional Research Ethics Committee of the Universidad Privada San Juan Bautista with resolution No. 0066-2023-CIEI-UPSJB. All participants (implant specialists and general dentists) provided written informed consent prior to participation.

## Results

The intraexaminer observation of general dentists and dental implant specialists revealed significant "almost perfect" agreement (p &lt;0.001), with the minimum and maximum values of Cohen's kappa index being similar in both groups at 0.82 (95% CI: 0.71-0.93) and 0.95 (95% CI: 0.89-1.00), respectively. Intraexaminer agreement (n = 25): median observed agreement P was 90.0% (IQR: 86.0%-92.0%); median PABAK was 0.80 (IQR: 0.72-0.84). By subgroup, general dentists (n = 12) showed median P (Observed agreement percentage) = 89.0% (IQR: 85.5%-92.0%) and median PABAK = 0.78 (IQR: 0.71-0.84); implant specialists (n = 13) showed median P = 90.0% (IQR: 86.0%-94.0%) and median PABAK = 0.80 (IQR: 0.72-0.88) (Table 2).

Table 2

The interexaminer observation of general dentists revealed "substantial" to "almost perfect" agreement that was statistically significant (p &lt; 0.001). The minimum and maximum values of the Fleiss's kappa index were 0.65 (95% CI: 0.61-0.68) and 0.95 (95% CI: 0.92-0.98), respectively. In general, the classification proposed showed "almost perfect" interexaminer agreement ( = 0.81, 95% CI: 0.80-0.82) (p &lt; 0.001), Po = 82.8%, AC1 (chance-adjusted agreement coefficient) = 0.82 (Table 3).

Table 3

The interexaminer observation of dental implant specialists revealed "substantial" to "almost perfect" agreement that was statistically significant (p &lt; 0.001). The minimum and maximum values of the Fleiss's kappa index were 0.71 (95% CI: 0.68-0.74) and 0.97 (95% CI: 0.94-1.00), respectively. In general, the proposed classification exhibited "almost perfect" interexaminer agreement ( = 0.89, 95% CI: 0.88 - 0.90) (p &lt; 0.001), Po = 90.0%, AC1 = 0.90 (Table 4).

Table 4

Finally, the interexaminer observation of all general dentists and dental implant specialists revealed statistically significant "substantial" to "almost perfect" agreement (p &lt; 0.001). The minimum and maximum values of Fleiss' were 0.71 (95% CI: 0.69-0.72) and 0.96 (95% CI: 0.95-0.98), respectively. In general, the proposed classification showed "almost perfect" interexaminer agreement ( = 0.84; 95% CI: 0.84-0.85; p &lt; 0.001), Po = 85.8%, AC1 = 0.85 (Table 5).

Table 5

**Figure 4:**
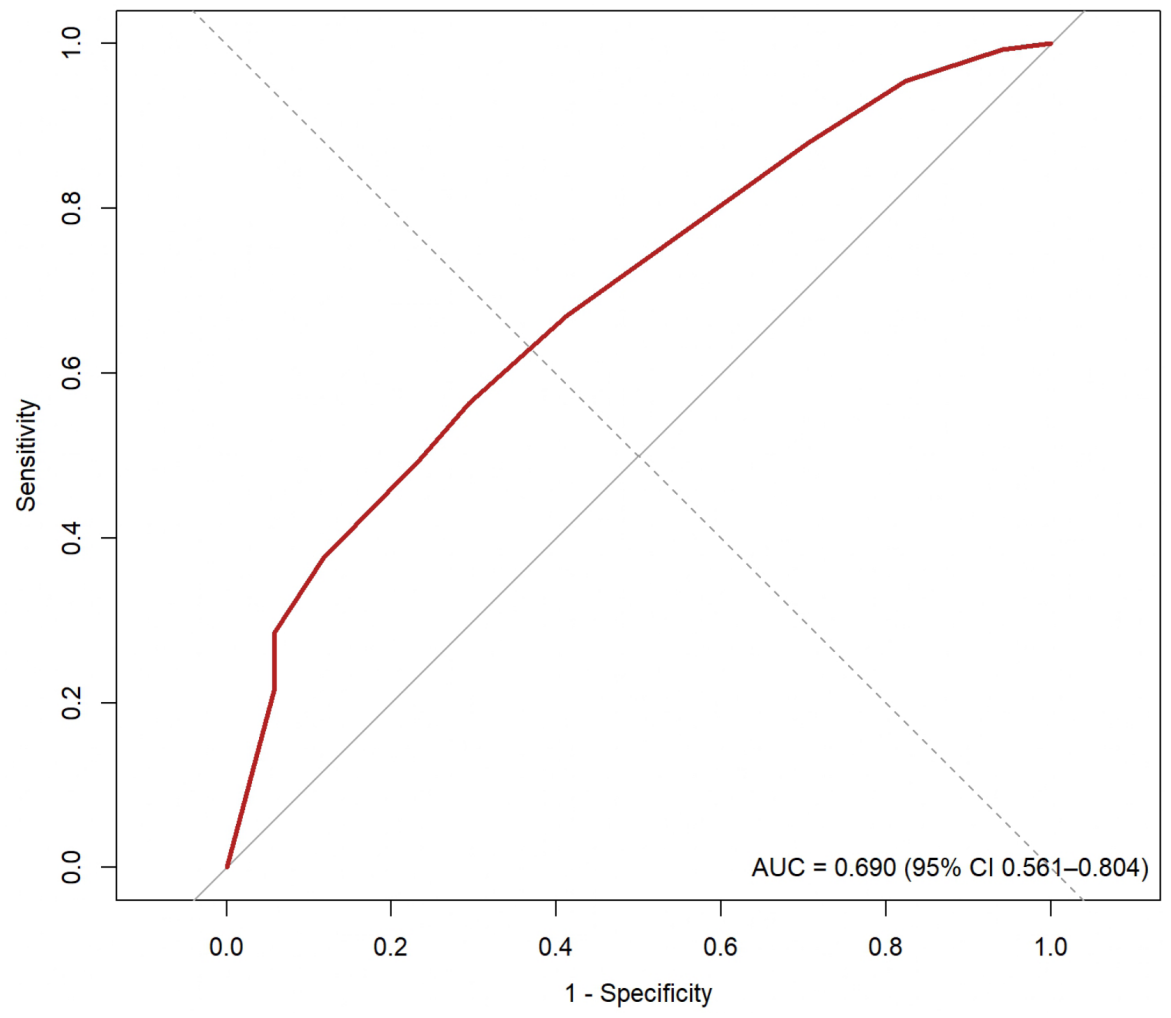
Receiver Operating Characteristic curve.

A retrospective cohort of 150 curves was followed from the time of implant placement for 4-6 months to evaluate the success rate (osseointegration). Most classes exceeded 85% success; II-A reached 96.7% and III-D 100%. In contrast, III-F (50%) and III-C (71.4%) showed the lowest performance, concentrating the highest proportion of failures. The 14-class specification yielded AUC = 0.69 (95% CI: 0.56-0.81) and Brier = 0.095, indicating moderate discriminative ability. Global calibration showed slight overestimation (intercept 0.10) and a slope of 1.19, reflecting limited dispersion of predicted probabilities (Table 6, Fig. 4).

Table 6

4

## Discussion

Preoperative assessment of bone quality is essential for surgical planning of dental implants and for achieving adequate primary stability (6). Although the final insertion torque is a critical factor for osseointegration, by itself, it does not predict the loading protocol or the biological stability that ultimately determines success. Accurate prediction requires analysis of the entire torque-time curve along the insertion path. In our study, the classification based on the progressive insertion torque value showed high intra- and inter-rater reliability for curve interpretation; however, the study was not designed to establish clinical efficacy or to define prosthetic loading thresholds. Torque-time signatures can be influenced by multiple factors-bone quality, trabecular thickness, marrow-space size, cortical thickness, the compressive surgical protocol (underdrilling by 0.2, 0.5, or 0.8 mm), implant macrodesign, length and diameter, surface treatment-related friction, and bone hardness influenced by systemic conditions, age, and sex, among others (6 , 17 - 19). The final insertion torque of a dental implant for type II-A bone (which has a good cortex and abundant cancellous bone with medium-thickness trabeculae and small medullary spaces) may be identical to that of type II-B bone (which has a good cortex and abundant cancellous bone but thin trabeculae and very wide medullary spaces) -for example, 45 Ncm; however, the progressive torque-time curve will be different, as the trabeculae of type II-A bone, which has a substantial amount of cortical and cancellous bone, are not very thin and have relatively small medullary spaces. In contrast, type II-B bone, which also has a substantial amount of cortical and cancellous bone, has trabeculae that are remarkably thin and have excessively wide medullary spaces. Consequently, it is not feasible to use the same loading protocol for both bone types. For instance, for type II-A bone, immediate loading could be applied within the first week of dental implant placement, early loading before two months, or conventional loading after two months. However, for type II-B bone, neither immediate nor early loading would be possible despite the attainment of the same final torque. Attaining final torques below 30 Ncm or above 50 Ncm through the use of implants of greater length or diameter does not indicate success or failure unless the progressive torque curve associated with bone quality is analyzed. The 30- and 50-Ncm values were adopted pragmatically as conventional anchors to stratify final torque rather than to prescribe loading decisions. Given variability across implant systems and osteotomy protocols, values near these boundaries should be interpreted cautiously and may require platform-specific adjustment; future work will empirically refine these cut-offs against clinical references (e.g., loading eligibility) using distributional, ROC-based, and calibration analyses. The macrodesign of the implant, as well as its length and diameter, could not directly influence the insertion torque of the dental implant when associated with less compressive surgical protocols (6 , 20 , 21). The objective of the present study was to propose a classification system for primary stability of dental implants based on progressive insertion torque that aims to provide optimal parameters for the successful primary and secondary stability of dental implants, with high repeatability and reproducibility. The results demonstrated that this novel approach exhibited a high degree of repeatability, with "almost perfect" intra-examiner agreement that was statistically significant. The inter-examiner agreement was also found to be "almost perfect" and statistically significant, suggesting minimal interpretation bias. The degree of bone compression resulting from dental implant placement, which is determined by the type of curve, allows for the planning of loading protocols and the prediction of the biological response in the final phase of osseointegration, which is known as secondary stability. Deficiencies in bone quantity and quality represent significant risk factors for implant failure, as evidenced by prior studies (1 , 5). Identifying conditions that place patients at high risk of failure enables surgeons to make informed decisions and adapt treatment to optimize outcomes. Achieving primary stability in patients with poor bone density is challenging and associated with higher rates of implant failure (1 , 3). Areas with poor bone quality may fail to establish primary implant stability, which could result in early implant failure (1 , 3). Conversely, implant placement in highly corticalized bone with minimal cancellous bone can result in excessively high insertion torques, which can lead to overcompression osteitis, a condition in which osteoclastic activity often results in early implant loss (3 , 5 , 22). It is not currently possible to conclude that very high (80 Ncm) or very low (&lt;20 Ncm) torques indicate osseointegration failure because of limited evidence in this area (23). Consequently, it is essential to consider not only the final torque but also the progressive torque curve, which begins at 0 Ncm and rises according to cancellous bone architecture in its initial segment (thickness of the trabeculae and size of the medullary spaces). The torque curve may show peaks and valleys as the implant advances, followed by abrupt decreases when encountering wide medullary spaces and very thin trabeculae. Along this trajectory, maximum torque may occur at the end of placement (i.e., the final torque), yielding a completely ascending curve. In other instances, the maximum torque may be reached in a specific region of the insertion path, after which it may adopt a horizontal or even descending trajectory (2 , 6). The sample analyzed allowed classification of the insertion torque curve of dental implants into three types and six subtypes according to bone quality. This approach provides parameters that enhance the repeatability and reproducibility of the proposed classification, thereby facilitating bone-quality assessment and the application of appropriate loading protocols to achieve successful osseointegration. One of the most widely utilized classifications of bone quality globally is that proposed by Lekholm and Zarb in 1985. This classification divides bone types into four categories according to their hardness, which is inferred from relative radiodensity of the cortical and cancellous bone, as well as cortical thickness (24). However, this subjective method of bone classification is insufficient for clinical application, as it was based solely on images obtained by various analog tomography units with differing kilovoltages, milliampere settings, and film quality. In contrast, the classification proposed by Rosas et al. in 2022 employs high-resolution tomography with digital imaging sensors and AI-enabled equipment, which allows more precise observation of the thickness of the trabeculae and the size of the medullary spaces. This updated classification adds three subtypes to type II, two to type III, and increases the number of subtypes within types IV (regenerated) and V (pathologic bone). Similarly, the original classification has limited capacity to anticipate prosthetic loading protocols or biological (secondary) stability as a function of bone compression (Ncm) during implant insertion (6). The present study proposes three types of primary stability or mechanical locking according to the final torque achieved, which is directly related to the quality and quantity of cancellous and cortical bone. Type I corresponds to bones that achieved primary stability with high torques greater than 50 Ncm. Type II corresponds to moderate torques ranging from 30-49 Ncm. Type III corresponds to implants that achieved mechanical torque &lt;29 Ncm. The three types of bone quality support the development of prosthetic implant loading protocols. The analysis of the six subtypes of curves facilitates understanding of bone remodeling according to the influence of osteoclastic and osteoblastic cells in cancellous and cortical bone, which are stimulated by the degree of bone compression during the process of implant insertion (25 - 27). For these reasons, this novel classification may have clinical relevance, but findings should be interpreted cautiously. In our data, the highest number of implant losses (n=5) occurred in I-A (very high torques with an upward curve throughout), particularly in type I bone; by contrast, in lower-density bone types, high torque did not coincide with loss in this sample. Two additional losses were observed in III-E (very low torque, flat curve) within type IV bone. Given the moderate discriminative performance and modest, uneven counts by subtype, these patterns are exploratory and require confirmation. If validated, the classification could inform decisions under conventional, early, or immediate loading and in both native and regenerated bone; however, generalizability across implant macrodesigns, lengths, and diameters should be established through external, multiplatform validation with possible recalibration. Furthermore, this classification was reproducible with a high degree of reliability, as evidenced by the calibration process with 13 dental implant specialists and 12 general dentists according to Fleiss' and Cohen's , which yielded "almost perfect" agreement (6 , 14 , 15). A limitation is the limited body of literature for direct comparisons. In addition, all torque-time curves were obtained using a single motor and a single implant system, under a fixed osteotomy protocol, and were performed by a single operator; although this approach reduced technical variability for reliability assessment, it also limits external validity. Because torque and ISQ measure different constructs (frictional locking vs resonance), correlation studies of these variables are required. This technique, which records progressive torque curves, is novel but increasingly accessible in academic and specialty settings. Prospective studies are needed to identify curve shapes as a function of variables influencing primary stability; to evaluate the predictive validity of the classification (AUC, ORs, calibration) and its performance in larger cohorts; and to compare intra- and inter-examiner calibration between novice and experienced clinicians. Regarding generalizability, the classification was developed and calibrated with a single system featuring a conical macrodesign; its performance across other macrodesigns (e.g., cylindrical bodies, different thread profiles) and extreme diameters should be assessed in multicenter, multiplatform validations, with possible recalibration of subtype thresholds.

## Conclusions

The classification of primary stability based on the progressive insertion torque value in edentulous maxillary ridges showed high intraexaminer and interexaminer reliability, supporting consistent interpretation of torque-time curves. However, the study was not designed to establish clinical efficacy or define prosthetic loading thresholds. Although bone compression is mechanistically linked to secondary stability, its predictive performance was not evaluated here. Prospective studies are needed to evaluate predictive validity and assess the clinical impact of the proposed classification.

## Figures and Tables

**Table 1:** Valuation of the kappa index.

kappa	Matching strength
0.00	Poor
0.01 - 0.20	Slight
0.21 - 0.40	Fair
0.41 - 0.60	Moderate
0.61 - 0.80	Substantial
0.81 - 1.00	Almost perfect

1

**Table 2:** Intraexaminer agreement in general dentists and dental implant specialists according to the proposed classification of bone quality considering the insertion torque of the dental implant.

Examiners	f	k	SE	95% CI		Statistical	*p	P0 (%)	PABAK
				LL	UL				
General dentists									
Examiner 1	50	0.822	0.057	0.710	0.934	18.067	<0.001	84.0	0.68
Examiner 2	50	0.911	0.043	0.828	0.994	19.957	<0.001	92.0	0.84
Examiner 3	50	0.866	0.051	0.766	0.966	18.597	<0.001	88.0	0.76
Examiner 4	50	0.933	0.037	0.860	1.000	20.271	<0.001	94.0	0.88
Examiner 5	50	0.889	0.047	0.797	0.981	19.223	<0.001	90.0	0.80
Examiner 6	50	0.823	0.056	0.712	0.933	18.509	<0.001	84.0	0.68
Examiner 7	50	0.845	0.054	0.738	0.951	18.516	<0.001	86.0	0.72
Examiner 8	50	0.911	0.042	0.828	0.994	20.135	<0.001	92.0	0.84
Examiner 9	50	0.889	0.047	0.797	0.981	19.424	<0.001	90.0	0.80
Examiner 10	50	0.823	0.057	0.711	0.935	18.311	<0.001	84.0	0.68
Examiner 11	50	0.844	0.054	0.738	0.950	18.396	<0.001	86.0	0.72
Examiner 12	50	0.956	0.031	0.895	1.000	20.930	<0.001	96.0	0.92
Dental implant specialists									
Examiner 13	50	0.888	0.047	0.796	0.981	19.076	<0.001	90.0	0.80
Examiner 14	50	0.845	0.054	0.739	0.951	18.535	<0.001	86.0	0.72
Examiner 15	50	0.823	0.057	0.712	0.935	18.456	<0.001	84.0	0.68
Examiner 16	50	0.867	0.051	0.767	0.966	18.951	<0.001	88.0	0.76
Examiner 17	50	0.844	0.054	0.739	0.949	18.402	<0.001	86.0	0.72
Examiner 18	50	0.866	0.051	0.766	0.966	18.639	<0.001	88.0	0.76
Examiner 19	50	0.933	0.037	0.860	1.000	20.418	<0.001	94.0	0.88
Examiner 20	50	0.889	0.047	0.797	0.981	19.166	<0.001	90.0	0.80
Examiner 21	50	0.956	0.031	0.895	1.000	20.760	<0.001	96.0	0.92
Examiner 22	50	0.933	0.037	0.860	1.000	19.985	<0.001	94.0	0.88
Examiner 23	50	0.889	0.047	0.797	0.981	19.470	<0.001	90.0	0.80
Examiner 24	50	0.845	0.054	0.740	0.951	18.790	<0.001	86.0	0.72
Examiner 25	50	0.956	0.031	0.895	1.000	20.973	<0.001	96.0	0.92

f: absolute frequency of Implant insertion torque curve in N•cm; k: Cohen’s Kappa Index; SE: Standard Error; 95% CI: 95% confidence interval; LL: lower limit; UL: upper limit; *Chi-square, p-value< 0.05 (significant association). Po: Observed agreement percentage; PABAK = Prevalence-Adjusted, Bias-Adjusted Kappa.

**Table 3:** Interexaminer agreement in general dentists according to the proposed classification of bone quality considering the insertion torque of the dental implant.

Classification Proposal	f	k	SE	95% CI		*p
				LL	UL	
I-A	111	0.95	0.02	0.92	0.98	<0.001
I-B	9	0.72	0.02	0.69	0.76	<0.001
I-C	10	0.82	0.02	0.78	0.85	<0.001
I-D	13	0.76	0.02	0.73	0.80	<0.001
II-A	83	0.77	0.02	0.74	0.81	<0.001
II-B	75	0.84	0.02	0.80	0.87	<0.001
II-C	29	0.65	0.02	0.61	0.68	<0.001
II-D	30	0.66	0.02	0.62	0.69	<0.001
III-A	35	0.74	0.02	0.71	0.78	<0.001
III-B	38	0.71	0.02	0.68	0.75	<0.001
III-C	42	0.76	0.02	0.73	0.80	<0.001
III-D	62	0.87	0.02	0.84	0.90	<0.001
III-E	29	0.79	0.02	0.76	0.83	<0.001
III-F	34	0.88	0.02	0.85	0.92	<0.001
Total	600	0.81	0.01	0.80	0.82	<0.001

f: absolute frequency of concordance; k: Fleiss Kappa Index; SE: Standard Error; LL: Lower Limit; UL: Upper Limit; 95% CI: 95% Confidence Interval; *Chi-square, p-value< 0.05 (significant association). Po: Observed agreement percentage = 82.8%; AC1: chance-adjusted agreement coefficient = 0.82.

**Table 4:** Interexaminer agreement in dental implant specialists according to the proposed classification of bone quality considering the insertion torque of the dental implant.

Classification proposal	f	k	SE	95% CI		*p
				LL	UL	
I-A	120	0.97	0.02	0.94	1.00	<0.001
I-B	13	0.71	0.02	0.68	0.74	<0.001
I-C	12	0.92	0.02	0.88	0.95	<0.001
I-D	17	0.77	0.02	0.74	0.80	<0.001
II-A	87	0.92	0.02	0.89	0.95	<0.001
II-B	94	0.89	0.02	0.86	0.93	<0.001
II-C	27	0.83	0.02	0.80	0.86	<0.001
II-D	39	0.85	0.02	0.81	0.88	<0.001
III-A	35	0.89	0.02	0.86	0.92	<0.001
III-B	32	0.76	0.02	0.73	0.80	<0.001
III-C	37	0.83	0.02	0.80	0.86	<0.001
III-D	64	0.92	0.02	0.89	0.95	<0.001
III-E	38	0.85	0.02	0.81	0.88	<0.001
III-F	35	0.91	0.02	0.88	0.94	<0.001
Total	650	0.89	0.01	0.88	0.90	<0.001

f: absolute frequency of concordance; k: Fleiss Kappa Index; SE: Standard Error; LL: Lower Limit; UL: Upper Limit; 95% CI: 95% Confidence Interval; *Chi-square, p-value<0.05 (significant association). Po: Observed agreement percentage = 90.0%; AC1: chance-adjusted agreement coefficient = 0.90.

**Table 5:** Interexaminer agreement in general dentists and dental implant specialists according to the proposed classification of bone quality considering the insertion torque of the dental implant.

Classification proposal	f	k	SE	95% CI		*p
				LL	UL	
I-A	231	0.96	0.01	0.95	0.98	<0.001
I-B	22	0.72	0.01	0.70	0.73	<0.001
I-C	22	0.87	0.01	0.86	0.89	<0.001
I-D	30	0.77	0.01	0.75	0.79	<0.001
II-A	170	0.84	0.01	0.82	0.86	<0.001
II-B	169	0.86	0.01	0.84	0.87	<0.001
II-C	56	0.71	0.01	0.69	0.72	<0.001
II-D	69	0.74	0.01	0.73	0.76	<0.001
III-A	70	0.81	0.01	0.79	0.82	<0.001
III-B	70	0.72	0.01	0.71	0.74	<0.001
III-C	79	0.79	0.01	0.77	0.81	<0.001
III-D	126	0.89	0.01	0.88	0.91	<0.001
III-E	67	0.81	0.01	0.79	0.82	<0.001
III-F	69	0.89	0.01	0.88	0.91	<0.001
Total	1250	0.84	0.00	0.84	0.85	<0.001

f: absolute frequency of concordance; k: Fleiss Kappa Index; SE: Standard Error; LL: Lower Limit; UL: Upper Limit; 95% CI: 95% Confidence Interval; *Chi-square, p-value< 0.05 (significant association). Po: Observed agreement percentage = 85.8%; AC1: chance-adjusted agreement coefficient = 0.85.

**Table 6:** Table Osseointegration by classification in a cohort of 150 curves to assess success or failure at 4–6 months.

Classification proposal	Osseointegration			
	Success		Failure	
	f	%	f	%
I-A	28	84.8	5	15.2
II-A	29	96.7	1	3.3
III-A	12	92.3	1	7.7
II-B	9	90.0	1	10.0
III-B	14	87.5	2	12.5
II-C	5	83.3	1	16.7
III-C	5	71.4	2	28.6
II-D	5	83.3	1	16.7
III-D	9	100.0	0	0
III-E	16	88.9	2	11.1
III-F	1	50.0	1	50.0

6

## Data Availability

The datasets used and/or analysed during this study can be obtained from the corresponding author (josecarlos.rosas@upsjb.edu.pe).

## Abbreviations

CI: Confidence Interval; LL: Lower Limit; SE: Standard Error; STROBE: STrengthening the Reporting of OBservational studies in Epidemiology; SPSS: Statistical Package for the Social Sciences; UL: Upper Limit.

## References

[1] Chrcanovic BR, Albrektsson T, Wennerberg A (2017). Bone quality and quantity and dental implant failure: a systematic review and meta-analysis. Int J Prosthodont.

[2] Al-Ekrish A, Widmann G, Alfadda S (2018). Revised, computed tomography-based Lekholm and Zarb jawbone quality classification. Int J Prosthodont.

[3] Goiato MC, dos Santos DM, Santiago JF, Moreno A, Pellizzer EP (2014). Longevity of dental implants in type IV bone: a systematic review. Int J Oral Maxillofac Surg.

[4] Castellano-Cosano L, Rodriguez-Perez A, Spinato S, Wainwright M, Machuca-Portillo G, Serrera-Figallo MA (2019). Descriptive retrospective study analyzing relevant factors related to dental implant failure. Med Oral Patol Oral Cir Bucal.

[5] Chrcanovic BR, Albrektsson T, Wennerberg A (2014). Reasons for failures of oral implants. J Oral Rehabil.

[6] Rosas-Díaz JC, Córdova-Limaylla NE, Palomino-Zorrilla JJ, Guerrero ME, Carreteros R, Cervantes-Ganoza LA (2022). Repeatability and Reproducibility of a Modified Lekholm and Zarb Bone Quality Classification Based on Cone Beam Computed Tomography: An Observatsion Study. J Int Soc Prev Community Dent.

[7] Shemtov-Yona K (2021). Quantitative assessment of the jawbone quality classification: a meta-analysis study. PLoS One.

[8] Tettamanti L, Andrisani C, Bassi MA, Vinci R, Silvestre-Rangil J, Tagliabue A (2017). Immediate loading implants: review of the critical aspects. Oral Implantol (Rome).

[9] Andersen OZ, Bellón B, Lamkaouchi M, Brunelli M, Wei Q, Procter P (2023). Determining primary stability for adhesively stabilized dental implants. Clin Oral Investig.

[10] Norton MR (2017). The influence of low insertion torque on primary stability, implant survival, and maintenance of marginal bone levels: a closed-cohort prospective study. Int J Oral Maxillofac Implants.

[11] Barros LA, da Silva CF, Camargos GV, de Oliveira GJ, Barros-Filho LA (2022). In vitro evaluation of the influence of bone cortical thickness on the primary stability of conventional- and short-sized implants. J Clin Exp Dent.

[12] Yang B, Irastorza-Landa A, Heuberger P, Ploeg HL (2022). Analytical model for dental implant insertion torque. J Mech Behav Biomed Mater.

[13] Trisi P, Berardi D, Paolantonio M, Spoto G, D’Addona A, Perfetti G (2013). Primary stability, insertion torque, and bone density of conical implants with internal hexagon: is there a relationship?. J Craniofac Surg.

[14] Landis JR, Koch GG (1977). The measurement of observer agreement for categorical data. Biometrics.

[15] Cerda J, Villarroel L (2008). Evaluation of the interobserver concordance in pediatric research: the Kappa coefficient. Rev Chil Pediatr.

[16] (2013). Declaration of Helsinki: ethical principles for medical research involving human subjects. JAMA.

[17] Rosas-Díaz JC, Malpartida-Carrillo V, Córdova-Limaylla NE, Guerrero ME, Palomino-Zorrilla JJ, Cervantes-Ganoza LA (2022). Resonance Frequency Analysis Mapping During Implant Healing Using a Nanostructured Hydroxyapatite Surface. J Int Soc Prev Community Dent.

[19] Li H, Liang Y, Zheng Q (2015). Meta-Analysis of Correlations Between Marginal Bone Resorption and High Insertion Torque of Dental Implants. Int J Oral Maxillofac Implants.

[20] Baldi D, Lombardi T, Colombo J, Cervino G, Perinetti G, Di Lenarda R, Stacchi C (2018). Correlation between insertion torque and implant stability quotient in tapered implants with knife-edge thread design. Biomed Res Int.

[21] Gómez-Polo M, Ortega R, Gómez-Polo C, Martín C, Celemín A, Del Río J (2016). Does length, diameter, or bone quality affect primary and secondary stability in self-tapping dental implants?. J Oral Maxillofac Surg.

[22] Ramesh R, Sasi A, Mohamed SC, Joseph SP (2024). “Compression Necrosis” - A Cause of Concern for Early Implant Failure? Case Report and Review of Literature. Clin Cosmet Investig Dent.

[23] Palomino-Zorrilla JJ, Córdova-Limaylla NE, Rosas-Díaz JC, Cayo-Rojas CF, Cervantes-Ganoza LA, Guerrero ME (2024). Jawbone quality classification in dental implant planning and placement studies. A scoping review. J Int Soc Prev Community Dent.

[24] Rosa C, Bento V, Duarte N, Sayeg J, Santos T, Pellizzer E (2024). Do dental implants installed in different types of bone (I, II, III, IV) have different success rates? A systematic review and meta-analysis. Saudi Dent J.

[25] Rosas J, Guerrero ME, Cordova N, Galindo M, García M, Cayo C (2024). The influence of the degree of dental implant insertion compression on primary stability measured by resonance frequency and progressive insertion torque: in vitro study. Biomedicines.

[26] Rosas J, Guerrero ME, Galindo M, García M, Espinoza E, Cayo C (2024). The importance of bone quality diagnostics in preventing displacement of dental implants within the mandibular body: a case report. J Int Soc Prev Community Dent.

[27] Rosas-Díaz J, Guerrero ME, Castillo-Andamayo D, Galindo-Gómez M, García-Luna M, Cervantes-Ganoza L (2024). Importance of local and systemic factors in preventing implant displacement in the mandibular body: a scoping review of existing literature. BMC Oral Health.

